# Synthesis and Characterization of New Ethylenediamine Platinum(IV) Complexes Containing Lipophilic Carboxylate Ligands

**DOI:** 10.1155/MBD.1995.57

**Published:** 1995

**Authors:** M. Galanski, B. K. Keppler

**Affiliations:** 1 Anorganisch-Chemisches Institut der Universität Heidelberg Im Neuenheimer Feld 270 Heidelberg D - 69120 Germany

## Abstract

A series of new ethylenediamine (en) platinum(IV) complexes of the type Pt^(IV)^enX_2_A_2_, with X_2_  = cyclobutane-1,1-dicarboxylato (CBDCA), dichloro or bis(decanoato) and A = acetato, dodecanoato, tetradecanoato, hexadecanoato, octadecanoato, adamantanecarboxylato (Ad) or 3α, 12α-diformoxy-5β-cholato (DFCA) were synthesized and characterized by elemental analysis, infrared and NMR (^1^H  and  ^13^C) spectroscopic techniques. Previous platinum(IV) compounds were usually restricted to trans-dihydroxo or trans-dichloro platinum(IV) complexes. Recently trans-dicarboxylato platinum(IV) complexes with mainly acetate, trifluoracetate or short-chain carboxylate groups (<11 carbons) in the axial position have been described in the literature^[1,2,3]^. In this paper we report on the synthesis and characterization of a new class of ethylenediamine platinum(IV) compounds that have high lipophilic long-chain carboxylate ligands either in the axial or equatorial position. The platinum(IV) compounds with the lipophilic trans-carboxylate ligands in the axial position were prepared by acylation of the trans-dihydroxo platinum(IV) species using an acyl halide in the presence of pyridine. In contrast to previous publications^[1]^ the yields were excellent (up to 94%!).


### SYNTHESIS AND CHARACTERIZATION OF NEW ETHYLENEDIAMINE PLATINUM(IV) COMPLEXES CONTAINING LIPOPHILIC CARBOXYLATE LIGANDS

M. Galanski and B. K. Keppler*

* Anorganisch-Chemisches Institut der Universität Heidelberg, Im Neuenheimer Feld 270, D - 69120 Heidelberg, Germany

### Abstract

A series of new ethylenediamine (en) platinum(IV) complexes of the type $Pt^{(IV)}enX_2A_2$ , with $X_2$ = cyclobutane-1,1-dicarboxylato (CBDCA), dichloro or bis(decanoato) and A = acetato, dodecanoato, tetradecanoato, hexadecanoato, octadecanoato, adamantanecarboxylato (Ad) or 3α,12α-diformoxy-5β-cholato (DFCA) were synthesized and characterized by elemental analysis, infrared and NMR ($^1H$ and $^{13}C$) spectroscopic techniques.
Previous platinum(IV) compounds were usually restricted to trans-dihydroxo or trans-dichloro platinum(IV) complexes. Recently trans-dicarboxylato platinum(IV) complexes with mainly acetate, trifluoracetate or short-chain carboxylate groups (<11 carbons) in the axial position have been described in the literature[1, 2, 3].
In this paper we report on the synthesis and characterization of a new class of ethylenediamine platinum(IV) compounds that have high lipophilic long-chain carboxylate ligands either in the axial or equatorial position. The platinum(IV) compounds with the lipophilic trans-carboxylate ligands in the axial position were prepared by acylation of the trans-dihydroxo platinum(IV) species using an acyl halide in the presence of pyridine. In contrast to previous publications[1] the yields were excellent (up to 94% !).

### Introduction cis-Diamminedichloroplatinum(II) (cisplatin, CDDP) is widely used to treat various types of human cancer. Although it is effective against testicular tumors, ovarian carcinomas, bladder tumors and tumors of the head and neck, its use is limited by significant severe side-effects such as dose-dependent nephrotoxicity, ototoxicity, neurotoxicity, myelotoxicity, nausea and vomiting[4]. In an attempt to overcome these limitations, it is desirable to develop new platinum-based anticancer drugs with a broader spectrum of activity, reduced toxicities and an improved clinical effectiveness. Both acceptance and quality of life of cancer patients receiving a platinum-based chemotherapy could be further enhanced by the development of orally administrable platinum complexes.
One strategy to attain this could be to replace the labile chloro ligands in CDDP with other leaving groups e.g. bidentate dicarboxylates and/or to replace the non-leaving groups with bis(alkylamine), diamine or mixed ammine/amine ligands. Several second-generation platinum drugs have entered clinical trials over the last two decades[5], such as diammine-(cyclobutane-1,1-dicarboxylato)platinum(II) (carboplatin)[6], (1,1-diaminocyclohexane)oxalato-platinum(II) (oxaliplatin)[7] and the racemic mixture of cis-bis(neodecanoato)-trans-R,R-1,2-diaminocyclohexaneplatinum(II) (L-NDDP) entrapped in liposomes as drug carriers[8, 9].
Another promising strategy in the search for new platinum-based anticancer drugs could be to convert platinum(II) compounds into their platinum(IV) analogues. Cis-dichloro-trans-dihydroxo-cis-bis(isopropylamine)platinum(IV) (CHIP)[10] was the first platinum(IV) complex to enter clinical trials. Tetraplatin[11], a racemic mixture of the l-trans and d-trans isomers of tetrachloro-

(1,2-diamino-cyclohexane)platinum(IV), was the second. Previous platinum(IV) compounds were usually restricted to the trans-dichloro- or trans-dihydroxoplatinum(IV) species.
trans-Dicarboxylatoplatinum(IV) compounds have recently been described in the literature. This new class of complexes is very interesting with regard to the development of platinum anticancer drugs that can be orally administered. Clinical trials with bis(acetato)(ammine)dichloro(cyclohexylamine)platinum(IV) (JM216) started a short while ago. Preclinical work has shown that JM216 has comparable p.o. activity to i.v. administered cisplatin and carboplatin in a panel of four human ovarian carcinoma xenografts in vivo[12]. Furthermore, JM216 is well absorbed from the gastrointestinal tract and has toxic effects comparable to those of carboplatin[13].
Therefore we report here on the systematic synthesis of platinum(IV) compounds that have trans-carboxylate ligands in the axial position. The general formula of these platinum(IV) complexes is $Pt^{(IV)}enX_2A_2$, with $X_2$ = cyclobutane-1,1-dicarboxylato (CBDCA), dichloro or bis(decanoato) and A = acetato, dodecanoato, tetradecanoato, hexadecanoato, octadecanoato, adamantanecarboxylato (Ad) or 3α,12α-diformoxy-5β-cholato (DFCA). The compound with the trans-acetate ligand was prepared by the general synthetic procedure: reaction of the trans-dihydroxoplatinum(IV) species with acetic anhydride. The yield was 93%. The new class of ethylenediamine platinum(IV) compounds with high lipophilic carboxylate ligands in the axial position was not prepared by the conventional synthetic pathway. In contrast to previous publications we used an acyl halide in the presence of pyridine with very good yields.

### Materials and Methods

### Chemicals

Silversulfate, hydrogen peroxide and acetic anhydride were purchased from Merck while ethylenediamine was obtained from Roth, cyclobutane-1,1-dicarboxylic acid from EGA and potassiumtetrachloroplatinate from Degussa. All chemicals obtained from commercial suppliers were used as received. Water was used bidistilled while the required acyl halides were prepared by reaction of the corresponding carboxylic acid with thionyl chloride. Workup was carried out by general methods.

### Physical Measurements

Elemental analyses were performed in our own laboratories. IR spectra were recorded in KBr pellets in the range of 400-4400 $cm^{-1}$ on a Bruker IFS 66. NMR spectra were measured using a Bruker Ac 200 MHz spectrometer. NMR ($^1H$, $^{13}C$) spectra were measured in $[D_6]$DMSO, $CDCl_3$, $[D_6]$ acetone or $D_2O$ containing $[D_6]$ acetone as reference. The $^{195}Pt$ NMR spectrum was obtained in $CDCl_3$ and referenced using an external sample of $H_2PtCl_6$ in $D_2O$ at 0.0 ppm.

### Preparation of Platinum Complexes

The trans-dicarboxylatoplatinum(IV) compounds with chloro ligands in the equatorial position were synthesized as shown in Scheme 1.

$$K_2PtCl_4 + en \longrightarrow PtenCl_2 + 2\,KCl \qquad (1)$$

$$PtenCl_2 + H_2O_2 \longrightarrow PtenCl_2(OH)_2 \qquad (2)$$

$$PtenCl_2(OH)_2 + 2\,RCOCl \longrightarrow PtenCl_2(OCOR)_2 + 2\,HCl \qquad (3)$$

**Scheme 1.** Synthesis of platinum(IV) compounds with chloro ligands in the equatorial position and carboxylate ligands in the axial position.

$PtenCl_2$ was prepared according to Drew's method[14] and oxidized by hydrogen peroxide (reactions (1) and (2)). The treatment of the trans-dihydroxoplatinum(IV) species with an acyl halide in the presence of pyridine produced the trans-dicarboxylatoplatinum(IV) complex in very good yields (reaction (3)).

*$Pt^{(II)}enCl_2$.* An aqueous solution of $K_2PtCl_4$ (6.04 g, 15.56 mmol) was treated with ethylenediamine (882 mg, 14.68 mmol) and stirred for 6 hours at room temperature. The precipitate that formed was filtered, washed with water and recrystallized from DMF/methanol. Anal. Calc. for $C_2H_8Cl_2N_2Pt$: C, 7.37; H, 2.47; N, 8.59. Found: C, 7.54; H, 2.50; N, 8.60. Yield, 61%.

*$Pt^{(IV)}enCl_2(OH)_2$.* 15 ml of 30% hydrogen peroxide were added to a suspension of $Pt^{(II)}enCl_2$ (875 mg, 2.68 mmol) in 15 ml of bidistilled water. After stirring for 30 minutes at room temperature, the mixture was heated under reflux for 10 minutes. The suspension was cooled and the product was filtered, washed with water and dried under reduced pressure. Anal. Calc. for $C_2H_{10}Cl_2N_2O_2Pt$: C, 6.67; H, 2.80; N, 7.78. Found: C, 6.72; H, 2.78; N, 7.77. Yield, 76%.

*$Pt^{(IV)}enCl_2(OCO(CH_2)_{12}CH_3)_2$.* After suspending $Pt^{(IV)}enCl_2(OH)_2$ (515 mg, 1.43 mmol) in 25 ml of acetone, tetradecanoyl chloride (2.1g, 8.52 mmol) and pyridine (888 mg, 11.23 mmol) were added. After stirring overnight at room temperature, the mixture was gently refluxed for 6 hours. The suspension was cooled down to room temperature and 70 ml of water were added to hydrolize the excess of acyl halide. The product and the tetradecanoic acid were filtered, washed with water and dried in vacuo. The carboxylic acid was extracted with $CHCl_3$ and the final product was dried under reduced pressure. $Pt^{(IV)}enCl_2(OCO(CH_2)_{12}CH_3)_2$ was obtained as a white solid. Anal. Calc. see Table 1. Yield, 94%.

All trans-dicarboxylatoplatinum(IV) complexes with chloro ligands in the equatorial position were prepared in a manner similar to the one described above. The elemental analyses and yields are listed in Table 1.

The ethylenediamine platinum(IV) complexes containing carboxylate ligands both in the equatorial and axial position were prepared as shown in Scheme 2.

$$K_2PtCl_4 + en \longrightarrow PtenCl_2 + 2\,KCl \qquad (1)$$

$$PtenCl_2 + Ag_2SO_4 \longrightarrow Pten(SO_4)(H_2O) + 2\,AgCl \qquad (5)$$

$$Pten(SO_4)(H_2O) + 2\,NaX \longrightarrow PtenX_2 + Na_2SO_4 \qquad (6)$$

$$PtenX_2 + H_2O_2 \longrightarrow PtenX_2(OH)_2 \qquad (7)$$

$$PtenX_2(OH)_2 + CH_3COOCOCH_3 \longrightarrow PtenX_2(OCOCH_3)_2 \qquad (8a)$$

$$PtenX_2(OH)_2 + 2\,RCOCl \longrightarrow PtenX_2(OCOR)_2 + 2\,HCl \qquad (8b)$$

**Scheme 2.** Synthesis of platinum(IV) compounds with mixed carboxylate ligands.

$PtenCl_2$ was prepared as described above and activated by reaction with an equimolar amount of silversulfate (reaction (1) and (5)). The platinum(II) complexes with different leaving groups were produced by mixing $Pten(SO_4)(H_2O)$ with the in situ prepared sodium salts of the corresponding carboxylic acids (reaction (6)). The obtained platinum(II) carboxylates were oxidized by hydrogen peroxide (reaction (7)). The trans-bis(acetato)platinum(IV) compound was prepared by reaction of the trans-dihydroxoplatinum(IV) species with acetic anhydride (reaction (8a)), whereas the platinum(IV) complexes with the lipophilic carboxylate ligands in the axial position were synthesized by reaction of $PtenX_2(OH)_2$ with an acyl halide in the presence of pyridine (reaction (8b)).

**Table 1.** Elemental Analyses of Ethylenediamine Platinum(IV) Complexes

| | | %C | %H | %Cl | %N | %Pt | Yield (%) |
|---|---|---|---|---|---|---|---|
| $Pt^{(IV)}enCl_2(OCO(CH_2)_{10}CH_3)_2$ | calc. | 43.09 | 7.51 | 9.78 | 3.87 | 26.92 | 89 |
| | found | 43.19 | 7.62 | 9.74 | 3.98 | 26.65 | |
| $Pt^{(IV)}enCl_2(OCO(CH_2)_{12}CH_3)_2$ | calc. | 46.15 | 8.00 | 9.08 | 3.59 | 24.98 | 94 |
| | found | 46.35 | 8.01 | 9.29 | 3.59 | 24.89 | |
| $Pt^{(IV)}enCl_2(OCO(CH_2)_{14}CH_3)_2$ | calc. | 48.79 | 8.43 | 8.47 | 3.35 | 23.31 | 92 |
| | found | 49.03 | 8.54 | 8.55 | 3.42 | 23.34 | |
| $Pt^{(IV)}enCl_2(OCO(CH_2)_{16}CH_3)_2$ | calc. | 51.11 | 8.80 | 7.94 | 3.14 | 21.78 | 91 |
| | found | 51.31 | 8.93 | 8.21 | 3.26 | 21.78 | |
| $Pt^{(IV)}enCl_2(Ad)_2$ | calc. | 42.11 | 5.60 | 10.36 | 4.09 | 28.50 | 88 |
| | found | 42.01 | 5.63 | 10.31 | 4.06 | 28.57 | |
| $Pt^{(IV)}enCl_2(DFCA)_2$ | calc. | 53.11 | 7.10 | 5.81 | 2.29 | 15.97 | 72 |
| | found | 53.31 | 7.18 | 5.79 | 2.36 | 15.08 | |
| $Pt^{(IV)}en(CBDCA)(OCO(CH_2)_{12}CH_3)_2$ | calc. | 50.75 | 8.04 | ---- | 3.29 | 22.90 | 68 |
| | found | 50.87 | 7.95 | ---- | 3.50 | 22.65 | |
| $Pt^{(IV)}en(CBDCA)(OCO(CH_2)_{14}CH_3)_2$ | calc. | 52.90 | 8.44 | ---- | 3.18 | 21.48 | 92 |
| | found | 53.09 | 8.30 | ---- | 3.16 | 21.36 | |
| $Pt^{(IV)}en(CBDCA)(OCO(CH_2)_{16}CH_3)_2$ | calc. | 54.81 | 8.78 | ---- | 2.91 | 20.23 | 89 |
| | found | 54.45 | 8.70 | ---- | 3.06 | 20.02 | |
| $Pt^{(IV)}en(OCO(CH_2)_8CH_3)_2(OCOCH_3)_2$ | calc. | 43.63 | 7.32 | ---- | 3.91 | 27.25 | 93 |
| | found | 43.45 | 7.23 | ---- | 3.98 | 27.64 | |

*$Pt^{(II)}en(SO_4)(H_2O)$.* An aqueous solution of silversulfate (726 mg, 2.33 mmol) was added to $Pt^{(II)}enCl_2$ (800 mg, 2.45 mmol) in 100 ml of bidistilled water. After stirring for 15 hours in the dark at room temperature, silverchloride was removed by filtration. The yellow solution was evaporated to dryness and the residue was dried over phosphorus(V)oxide in vacuo. Anal. Calc. for $C_2H_{10}N_2O_5PtS$: C, 6.51; H, 2.73; N, 7.59. Found: C, 6.64; H, 2.78; N, 7.70. Yield, 97%.

*$Pt^{(II)}en(CBDCA)$.* Cyclobutane-1,1-dicarboxylic acid (144 mg, 2.26 mmol) was dissolved in 4.65 ml of 1N NaOH and stirred for 30 minutes at 50°C. After the addition of 835 mg (2.26 mmol) $Pt^{(II)}en(SO_4)(H_2O)$ in 35 ml of water, the mixture was stirred for two hours at 50°C and for 18 hours in the dark at room temperature. The produced platinum(II) complex was filtered, washed with acetone and dried in vacuo. Anal. Calc. for $C_8H_{14}N_2O_4Pt$: C, 24.19; H, 3.55; N, 7.05. Found: C, 24.15; H, 3.56; N, 7.04. Yield, 80%.

*$Pt^{(IV)}en(CBDCA)(OH)_2$.* The addition of 10 ml of 30% hydrogen peroxide to a suspension of 305 mg (768 μmol) $Pt^{(II)}en(CBDCA)$ in 15 ml of water produced a clear solution. After a few minutes, the white product began to precipitate. The suspension was stirred overnight at room temperature, filtered and washed with water. Anal. Calc. for $C_8H_{16}N_2O_6Pt$: C, 22.28; H, 3.74; N, 6.50; Pt, 45.23. Found: C, 22.37; H, 3.74; N, 6.50; Pt, 45.04. Yield, 58%.

*$Pt^{(IV)}en(CBDCA)(OCO(CH_2)_{12}CH_3)_2$.* The following trans-dicarboxylatoplatinum(IV) compounds with the cyclobutane-1,1-dicarboxylate ligand in the equatorial position were prepared in a manner similar to the one used in the case of $Pt^{(IV)}enCl_2(OCO(CH_2)_{12}CH_3)_2$. The elemental analyses and yields of these complexes are listed in Table 1.

*$Pt^{(II)}en(OCO(CH_2)_8CH_3)_2$.* Sodiumdecanoate, prepared in situ by mixing 3.54 ml of 1 N NaOH and 595 mg (3.45 mmol) of decanoic acid, was added to a solution of 638 mg (1.73 mmol) $Pt^{(II)}en(SO_4)(H_2O)$ in 10 ml of bidistilled water. After stirring for 30 minutes at 60°C, one hour at 45°C and two days in the dark at room temperature, the whole mixture was extracted with $CHCl_3$. The organic layer was dried over magnesiumsulfate and evaporated to dryness. The residue was extracted with acetone and the remaining solid was dried in vacuo. Anal. Calc. for $C_{22}H_{46}N_2O_4Pt$: C, 44.21; H, 7.76; N, 4.69; Pt, 32.64. Found: C, 44.37; H, 7.83; N, 4.72; Pt, 32.78. $^{13}C$=O, 181.94. $^{195}Pt$, -1736. Yield, 76%.

*$Pt^{(IV)}en(OCO(CH_2)_8CH_3)_2(OH)_2$.* 218 mg (365 μmol) $Pt^{(II)}en(OCO(CH_2)_8CH_3)_2$ were suspended in 6 ml of water and 12 ml of 30% hydrogen peroxide were added. The mixture was stirred for 30 minutes at room temperature and for 5 minutes at 50°C. After filtration the unreacted ethylenediamine platinum(II) species was extracted with $CHCl_3$. The dried product was obtained as a white solid. Anal. Calc. for $C_{22}H_{48}N_2O_6Pt$: C, 41.83; H, 7.66; N, 4.43; Pt, 30.88. Found: C, 42.08; H, 7.75; N, 4.41; Pt, 30.62. Yield, 23%.

*$Pt^{(IV)}en(OCO(CH_2)_8CH_3)_2(OCOCH_3)_2$.* Acetic anhydride (50 mg, 490 μmol) was added to a suspension of $Pt^{(IV)}en(OCO(CH_2)_8CH_3)_2$ (40 mg, 63 μmol) in 20 ml of acetone. After stirring overnight at room temperature, the mixture was heated for 2 hours at 50°C and cooled down to room temperature. 30 ml of water were added. After an overnight storage in the refrigerator, the white precipitate was filtered off and dried over phosphorus(V)oxide. Anal Calc. for $C_{26}H_{52}N_2O_8Pt$: C, 43.63; H, 7.32; N, 3.91; Pt, 27.25. Found: C, 43.45; H, 7.23; N, 3.98; Pt, 27.64. Yield, 93%.

### Results and Discussion

We have synthesized a new class of ethylenediamine platinum(IV) complexes containing lipophilic long-chain carboxylate ligands. The structural features of these complexes of the type $Pt^{(IV)}enX_2A_2$ with $X_2$ = two chloro, two carboxylato or one bidentate dicarboxylato ligands and A = carboxylato are illustrated in Figure 1.

The compounds were characterized by elemental analysis, IR and, if soluble enough, by NMR spectroscopy. The results of the elemental analyses are listed in Table 1. The theoretical values are in good agreement with the actual findings.

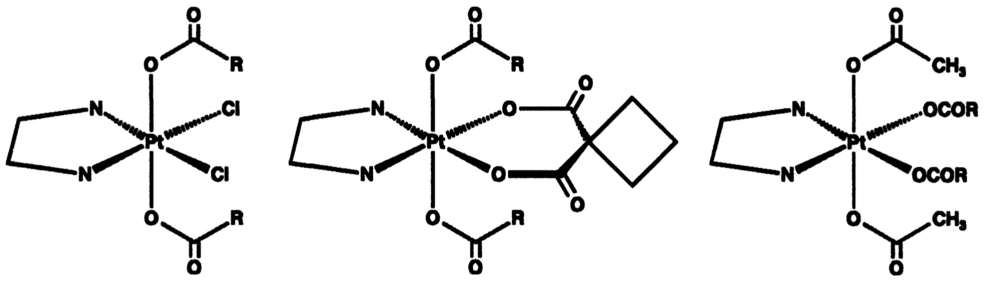

**Figure 1.** Structures of the synthesized ethylenediamine platinum(IV) complexes.

The IR spectroscopic data for the trans-dicarboxylatoplatinum(IV) compounds is listed in Table 2. In general, $Pt^{(II)}enCl_2$ and ethylenediamine platinum(II) compounds show two single sharp and resolvable N-H stretching absorptions. The platinum(IV) analogues with mixed dicarboxylate ligands showed one broad band whereas platinum(IV) complexes with chloro ligands in the equatorial and carboxylato ligands in the axial position displayed one single sharp peak in the region of 3204 $cm^{-1}$.

**Table 2.** Infrared Data for Ethylenediamine Platinum(IV) Complexes.

| | IR ($cm^{-1}$) | | |
|---|---|---|---|
| | ν(N-H) | $\nu_{as}$(COO) | $\nu_s$(COO) |
| $Pt^{(IV)}enCl_2(OCO(CH_2)_{10}CH_3)_2$ | 3204 | 1645 | 1374 |
| $Pt^{(IV)}enCl_2(OCO(CH_2)_{12}CH_3)_2$ | 3204 | 1644 | 1375 |
| $Pt^{(IV)}enCl_2(OCO(CH_2)_{14}CH_3)_2$ | 3203 | 1644 | 1372 |
| $Pt^{(IV)}enCl_2(OCO(CH_2)_{16}CH_3)_2$ | 3204 | 1645 | 1374 |
| $Pt^{(IV)}enCl_2(Ad)_2$ | 3204 | 1635 | 1329 |
| $Pt^{(IV)}enCl_2(DFCA)_2$ | 3206 | 1722, 1651 | 1367 |
| $Pt^{(IV)}en(CBDCA)(OCO(CH_2)_{12}CH_3)_2$ | 3179 | 1639 | 1345 |
| $Pt^{(IV)}en(CBDCA)(OCO(CH_2)_{14}CH_3)_2$ | 3181 | 1647 | 1348 |
| $Pt^{(IV)}en(CBDCA)(OCO(CH_2)_{16}CH_3)_2$ | 3180 | 1637 | 1346 |
| $Pt^{(IV)}en(OCO(CH_2)_8CH_3)_2(OCOCH_3)_2$ | 3233 | 1636 | 1362 |

For all trans-dicarboxylatoplatinum(IV) complexes one carbonyl vibration frequency in the region around 1636-1651 $cm^{-1}$ was observed. In addition, $Pt^{(IV)}enCl_2(DFCA)_2$ showed a very strong carbonyl absorption at 1722 $cm^{-1}$, which is assigned to the formoxy-groups of the 3α,12α-diformoxy-5β- cholato ligands. These values are in good agreement with those reported in the literature[3, 15, 16].

Almost all of the synthesized platinum(IV) compounds were insoluble or barely soluble in most of the common organic solvents. Only one $^1H$ NMR spectrum of $Pt^{(IV)}enCl_2(DFCA)_2$ and an $^1H$ and $^{13}C$ NMR spectrum of $Pt^{(IV)}enCl_2(Ad)_2$ and $Pt^{(IV)}en(OCO(CH_2)_8CH_3)_2(OCOCH_3)_2$ could be obtained. The $^{13}C$ resonance of the carbonyl group of $Pt^{(IV)}enCl_2(Ad)_2$ was observed at 186.5. Unfortunately, the $^{13}C$=O resonance of $Pt^{(IV)}en(OCO(CH_2)_8CH_3)_2(OCOCH_3)_2$ was too weak to be reported.

In conclusion, a series of new ethylenediamine platinum(IV) compounds containing lipophilic long-chain carboxylate ligands either in the axial or equatorial position have been synthesized and characterized. Moreover, the use of the unconventional synthetic pathway via an acyl halide produced yields up to 94%. The potentially useful trans-dicarboxylatoplatinum(IV) complexes will be tested with regard to their antitumor activity and oral administration possibilities.

### References

[1] C. M. Giandomenico, K. R. Harrap, P. M. Goddard, L. R. Kelland, S. E. Morgan, *Platinum and Other Metal Coordination Complexes in Cancer Chemotherapy*, S. B. Howell (Ed), Plenum Press, New York, 1991, 93

[2] L. R. Kelland, B. A. Murrer, G. A. Abel, C. M. Giandomenico, P. Mistry, K. R. Harrap, *Cancer*

*Res.* 1992, **52**, 822
[3] A. R. Khokhar, Y. Deng, Y. Kido, Z. H. Siddik, *J. Inorg. Biochem.* 1993, **50**, 79
[4] D. D. Von Hoff, R. Schilsky, C. M. Reichert, R. L. Reeddick, M. Rozencweig, R. C. Young, F.M. Muggia, *Cancer Treat. Rep.* 1979, **63**, 1527
[5] L. R. Kelland, S. J. Clarke, M. J. McKeage, *Plat. Met. Rev.* 1992, **36**(4), 178
[6] A. H. Calvert, S. J. Harland, D. R. Newell, Z. H. Siddik, A. C. Jones, T. J. McElwain, R. K. Shanti, E. Wiltshaw, I. E. Smith, J. W. Baker, M. J. Peckham, K. R. Harrap, *Cancer Chemother. Pharmacol.* 1982, **9**, 140
[7] J. L. Misset, Y. Kidani, J. Gastiaburu, C. Jasmin, F. Levi, N. Boughattas, G. Lemaigre, J. P. Caussanel, S. Brienza, B. Kim Triana, E. Goldschmidt, M. Musset, R. Y. Mauvernay, G. Mathe, *Platinum and Other Metal Coordination Compounds in Cancer Chemotherapy*, S. B. Howell (Ed), Plenum Press, New York, 1991, 369
[8] A. R. Khokhar, S. Al-Baker, R. Perez-Soler, *Anti-Cancer Drug Design* 1988, **3**, 177
[9] R. Perez-Soler, G. Lopez-Berestein, J. Lautersztain, S. Al-Baker, K. Francis, D. Macias-Kiger, M. N. Rabere, A. R. Khokhar, *Cancer Res.* 1990, **50**, 4254
[10] P. Ribaud, J. Gouveia, J. L. Misset, G. Mathe, *Oncology* 1986, **43**, 78
[11] M. C. Christian, D. Spriggs, K. D. Tutsch, T. O'Rourke, D. D. VonHoff, J. L. Jacob, E. Reed, *Platinum and Other Metal Coordination Compounds in Cancer Chemotherapy*, S. B. Howell (Ed), Plenum Press, New York, 1991, 453
[12] L. R. Kelland, M. Jones, J. J. Gwynne, M. Valenti, B. A. Murrer, C. F. J. Barnard, J. F. Vollano, C. M. Giandomenico, M. J. Abrahms, K. R. Harrap, *Int. J. Oncol.* 1993, **2**,1043
[13] K. R. Harrap, B. A. Murrer, C. Giandomenico, S. E. Morgan, L. R. Kelland, M. Jones, P. M. Goddard, J. Schurig, *Platinum and Other Metal Coordination Compounds in Cancer Chemotherapy*, S. B. Howell (Ed), Plenum Press, New York, 1991, 391
[14] H. D. K. Drew, *J. Chem. Soc.* 1932, 2328
[15] A. R. Khokhar, Y. Deng, J. Coord. Chem. 1992, **25**, 349
[16] A. R. Khokhar, Y. Deng, S. Al-Baker, M. Yoshida, Z. H. Siddik, J. Inorg. Biochem. 1993, **51**, 677

**Received: September 13, 1994 - Accepted in camera-ready format: September 26, 1994**

